# Automated Ribotyping and Pulsed-Field Gel Electrophoresis for Rapid Identification of Multidrug-Resistant *Salmonella* Serotype Newport

**DOI:** 10.3201/eid0904.020423

**Published:** 2003-04

**Authors:** John Fontana, Alison Stout, Barbara Bolstorff, Ralph Timperi

**Affiliations:** * Massachusetts Department of Public Health, Jamaica Plain, Massachusetts, USA

## Abstract

In a series of 116 *Salmonella enterica* Newport isolates that included 64 multidrug-resistant (MDR) isolates, automated ribotyping and pulsed-field gel electrophoresis (PFGE) discriminated MDR *S*. Newport with a sensitivity of 100% and 98% and specificity of 76% and 89%, respectively. Clustering of PFGE patterns (but not ribotyping) linked human and bovine cases. Automated ribotyping rapidly identified the MDR strain, and PFGE detected associations that aided epidemiologic investigations.

An eight-drug resistant strain of *Salmonella enterica* serotype Newport (multidrug-resistant [MDR] *S*. Newport) characterized by resistance to at least ampicillin, cephalothin, chloramphenicol, clavulanic acid, streptomycin, sulfamethoxazole, tetracycline, and ceftriaxone (intermediate to complete) recently identified by the National Antimicrobial Resistance Monitoring System (NARMS), Centers for Disease Control and Prevention (CDC) (*1*), is an example of the emergent problem of antibiotic resistance. However, only a few state laboratories routinely perform susceptibility testing on enteric organisms, particularly in a format comparable to that employed by NARMS. Rapid identification of drug resistance is critical for preventing and treating diseases and for epidemiologic analysis (*2*).

Pulsed-field gel electrophoresis (PFGE) testing is more common at state public health laboratories; 50 such laboratories participate in CDC’s PFGE surveillance program, PulseNet. In PulseNet, participants submit PFGE data on *E. coli* O157:H7, *Salmonella* spp., *Shigella* spp., and *Listeria monocytogenes* to a national database (*3*). Because PulseNet laboratories follow a standardized protocol, PFGE patterns can be compared reliably within the network and associations made promptly among isolates that may be few in number and widely separated geographically.

However, the clonal nature or inherent genetic variability of bacteria may limit the ability of PFGE either to link isolates or to detect relatedness among a set of isolates from a single outbreak (*4*). In some organisms, such as *Shigella* spp. and *Campylobacter* spp., PFGE patterns vary considerably, and differences in banding patterns can occur among isolates that are epidemiologically linked (*5*). In other organisms, such as *Salmonella* serotype Enteritidis, variability of PFGE patterns is limited, and banding patterns can be indistinguishable among isolates that are not epidemiologically linked. Often, supplemental methods are needed to detect an association among isolates because of their apparent clonal characteristics (*6*). Therefore, PFGE databases must contain patterns from a sufficient number of isolates of a species representative of circulating strains to enable accurate interpretation of relatedness.

In Massachusetts, *S.* Newport isolates analyzed by PFGE have shown a high degree of variability. Ribotyping has been reported to be less discriminatory than PFGE (*7*) but can provide information that identifies real associations undetected solely by PFGE analysis, particularly when epidemiologic data are limited (*8*). We evaluated the usefulness of PFGE and automated ribotyping, independently and together, to monitor and characterize the MDR strain of *S.* Newport in Massachusetts.

## The Study

Specimens of *Salmonella* spp. submitted to the State Laboratory Institute as pure cultures or isolates from fresh stool samples submitted in Meridian Para-Pak C & S medium (Meridian Bioscience, Inc., Cincinnati, OH) were serotyped by the Kaufmann-White scheme, according to CDC protocols (*9*). After an increase in the incidence of *S.* Newport , the Massachusetts Department of Public Health initiated enhanced surveillance for this bacterium. In December 2000, we posted patterns MA-JJP0034 and MA-JJP0070 on the PulseNet listserv and asked other states to determine detection of *S*. Newport (CDC PulseNet patterns JJP.X01.0014 and JJP.X01.0181, respectively). In response, Oklahoma, Minnesota, Maine, and Vermont posted similar or indistinguishable isolates. In January 2001, *S.* Newport was isolated from a stool specimen from an employee at a Massachusetts farm that also reported diarrheal illness in cows. Investigators from the Massachusetts Department of Public Health and the Massachusetts Department of Food and Agriculture, Bureau of Animal Health, with assistance from CDC, visited farms and auction houses to obtain stool specimens from cows and calves exhibiting diarrheal illness. Isolates identified as *S.* Newport were tested by PFGE and automated ribotyping, and for antimicrobial resistance.

All *S.* Newport isolates were tested for resistance to amikacin, ampicillin, amoxicillin/clavulanic acid, apramycin, cefoxitin, ceftiofur, ceftriaxone, cephalothin, chloramphenicol, ciprofloxacin, gentamicin, kanamycin, nalidixic acid, streptomycin, sulfamethoxazole, tetracycline, and trimethoprim/sulphamethoxazole by using the Trek Diagnostics Sensititre CMV1UIL (Trek Diagnostic Systems, Inc., Cleveland, OH) plate. Isolates categorized as MDR *S.* Newport were resistant to at least ampicillin, cephalothin, chloramphenicol, clavulanic acid, streptomycin, sulfamethoxazole, and tetracycline, and demonstrated complete or intermediate resistance to ceftriaxone. An *E. coli* strain, ATTC 25922, was used as a control organism during each susceptibility test run.

PFGE was performed on all *S*. Newport isolates, according to previously described methods (*10*). Automated ribotyping was performed by using the Riboprinter Microbial Characterization System (DuPont Qualicon, Wilmington, DE), according to the manufacturer’s protocol. Briefly, a sweep of pure *S.* Newport cells from each test isolate was suspended in a 12- x 75-mm Falcon tube in sample buffer to 20% transmittance in a Vitek Colorimeter (bioMérieux, Durhan, NC). Thirty microliters of each suspension was transferred to one well in an eight-well sample carrier and heat treated to 80°C for 30 min; 5 µL of lysis buffers A and B was then added. The sample carrier was then placed in the Riboprinter for automated processing, which included the following steps: DNA extraction, *Pvu*II restriction and electrophoresis through a 1% agarose gel, DNA transfer, and hybridization to a nylon membrane. The DNA was probed with a sulfonated 1,020-bp *E. coli rrn,* which was detected with alkaline phosphatase–labeled anti-sulfonated anti-DNA antibodies. Ribotype images were captured by a charge-coupled device camera and compared for similarity to images in a *Pvu*II database of 272 *Salmonella* patterns of 138 serotypes (*11*).

PFGE patterns were analyzed with Molecular Analyst Software Version 1.11 (Bio-Rad Laboratories, Hercules, CA) by using the Dice coefficient with a band position tolerance of 1.5%. This method considers only the presence or absence of a band. All *S*. Newport patterns were compared with pattern MA-JJP0034, the first pattern of MDR *S.* Newport that appeared in Massachusetts. Patterns with a similarity coefficient of >0.85 relative to pattern MA-JJP0034 were designated in the analysis as MDR *S.* Newport by PFGE (D. Boxrud, pers. comm.).

Ribotype patterns were analyzed by the Riboprinter software, using the Pearson coefficient against a database of unique patterns of *Salmonella* serotypes. In this study, the *S.* Newport ribotypes with a similarity >0.85. were included in the MDR ribogroup. Sensitivity and specificity of the PFGE and ribotyping methods to identify MDR *S*. Newport were calculated and compared to the test results obtained by serotyping and antibiotic sensitivity testing for the 130 *S.* Newport isolates (*12*).

## Conclusions

One hundred sixteen *S*. Newport isolates from 300 human specimens and 14 *S*. Newport isolates from 50 bovine specimens were identified by serotyping at the State Laboratory Institute during the study period; 64 isolates were identified as MDR *S*. Newport (50 [43%] of 116 human isolates and all 14 bovine isolates). In addition to the eight-drug resistance pattern of the MDR isolates, 11 of 50 human isolates and 11 of 14 bovine isolates were resistant to kanamycin.

**Figure 1:**
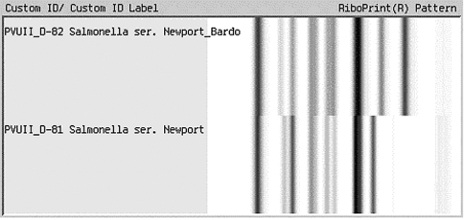
Ribotype patterns for *Salmonella* Newport.

Two ribotype patterns, D-81 and D-82, designated as *S*. Newport in the Riboprinter database, were associated with the 130 *S*. Newport serotyped isolates (Figure 1). The 64 MDR *S*. Newport isolates, 50 human isolates, and 14 bovine isolates, matched only to ribotype pattern D-81. Sixteen of 66 pansusceptible *S*. Newport human isolates also were identified as D-81 ribotype pattern. The remaining 50 pansusceptible *S*. Newport isolates were identified as D-82 ribotype pattern (Table). Using pattern D-81, ribotyping had a sensitivity of 100% and a specificity of 76% in identifying MDR *S*. Newport among *S*. Newport isolates.

**Table:** Sensitivity and specificity of PFGE versus. ribotyping in identifying MDR *Salmonella* Newport^a^

PFGE of *S.* Newport				Ribotyping of *S.* Newport			
PFGE pattern MA-JJP0034	MDR	Not MDR	Total	D-81 ribotype	MDR	Not MDR	Total
Yes^b^	63	7	70	Yes	64	16	80
No	1	57	58	No	0	50	50
	64	64^c^	128		64	66	130

^a^PFGE, pulsed-field gel electrophoresis; MDR, multidrug resistant. ^b^>85% relatedness to MA-JJP0034. ^c^Two pansusceptible isolates smeared by PFGE.

**Figure 2:**
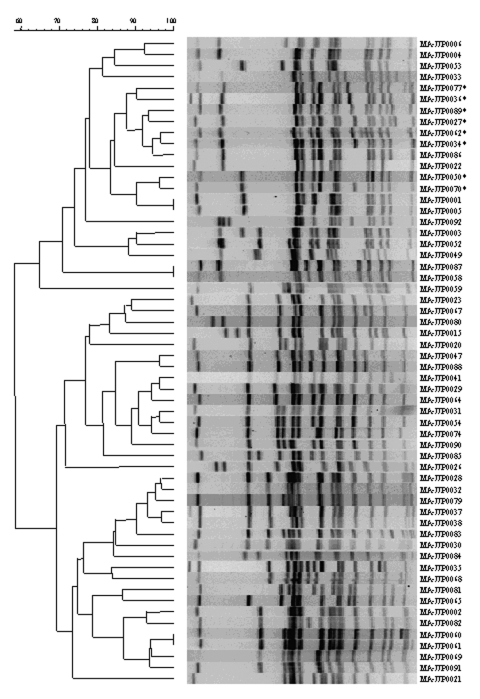
Dendrogram of unique pulsed-field gel electrophoresis patterns of *Salmonella* Newport. The 58 patterns represent all patterns received at the State Laboratory Institute during April 1999–April 2001. * indicates multidrug-resistant *S*. Newport patterns.

**Figure 3:**
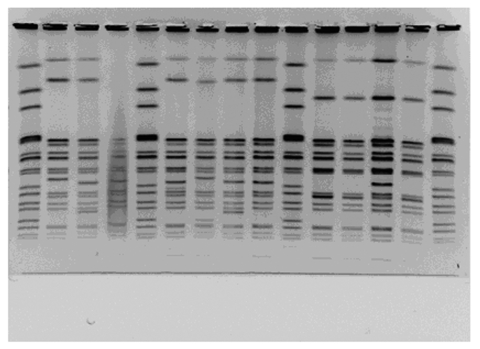
Pulsed-field gel electrophoresis patterns of *Xba*I-digested multidrug-resistant *Salmonella* Newport. Lane 2, pattern MA-JJP0036; lane 3, pattern MA-JJP 0027; lane 6, pattern MA-JJP0062; lane 7, pattern MA-JJP0077; lane 8, pattern MA-JJP0089; lane 9, pattern MA-JJP0034; lane 11, pattern MA-JJP0050; lane 13, pattern MA-JJP0070.

Among the 130 human and bovine *S*. Newport isolates were 58 PFGE patterns of *Xba*I digests (Figure 2). Eight PFGE patterns were associated with 64 MDR *S*. Newport isolates (Figure 3). Two of the 66 pansusceptible isolates smeared by PFGE were not interpretable, leaving 64 isolates for PFGE analysis. The most common PFGE pattern was MA-JJP0034, which appeared in 41 (64%) of 64 of the MDR *S*. Newport isolates. MA-JJP00015 was the most common pattern among the pansusceptible isolates, being found in 7 (11%) of 64 isolates. When the *S*. Newport isolates were analyzed against PFGE pattern 34, all MDR *S*. Newport isolates and 7 of 64 pansusceptible *S.* Newport isolates had a similarity coefficient of >0.85*.* Thus, PFGE had a sensitivity of 98% and a specificity of 89% when this pattern was used to screen this set of isolates.

The 64 MDR *S*. Newport, among a sample of 130 *S*. Newport isolates, were correctly identified by comparing the sensitivity and specificity of PFGE and automated ribotyping to conventional serotyping and antibiotic susceptibility test results. The sensitivity of each method was >98%. The specificity of PFGE and automated ribotyping was 89% and 76%, respectively, with no false-negative results, and 11% and 24%, respectively, of *S*. Newport isolates misidentified (false positive) as the MDR strain.

Automated ribotyping was a rapid means of subtyping *S*. Newport and distinguishing the MDR strain from non-MDR strains by using the Riboprinter *Pvu*II database. This capability is important because most often *Salmonella* isolates are submitted to state laboratories and characterized to the O antigen level, not the species level. Further, serotyping performed at a state public health laboratory to define the *Salmonella* species requires a minimum of 2 days, and for biphasic *Salmonella* species, such as *S.* Newport a minimum of 4 days (*9*). PFGE was useful in distinguishing associations within the isolates of *S.* Newport*,* which allowed for identification of potential epidemiologically related events, such as the PFGE pattern of MDR *S*. Newport common among a subset of bovine and human isolates. After a pure culture isolate is obtained, turnaround times for PFGE and ribotyping testing are approximately 24 h and 8 h, respectively; however, serotype identification often is required for interpretation of PFGE test results, depending on the organism, which can lengthen turnaround for final results by 1 or more days.

Both automated ribotyping and PFGE rely on existing databases of images from individual isolates to characterize DNA fingerprints. As data from more strains are added to databases, characterizing similarities or differences between isolates becomes easier. Because the fingerprint database of the PulseNet program is extensive and PFGE is more discriminatory that ribotyping, PFGE provides a more robust tool in characterizing the development of emerging pathogens.

These data suggest that PFGE and ribotyping can be used together to provide rapid identification of the MDR strain of *S*. Newport. These methods offer important capabilities for laboratory-based surveillance for public health purposes; however, automated ribotyping is very costly and should be used in a public health laboratory only if justified by appropriate cost-benefit analysis. Furthermore, these data support the need for strengthening public health laboratory infrastructure to facilitate early detection of infectious disease risks.
